# Endogenous Bioelectrical Modulation of Longevity-Associated and Inflammatory Signaling Pathways in Human Dermal Fibroblasts Following the REAC ACT-IBZ Protocol

**DOI:** 10.3390/life16040650

**Published:** 2026-04-12

**Authors:** Sara Cruciani, Vania Fontani, Arianna Rinaldi, Giuseppe Garroni, Diletta Serra, Salvatore Rinaldi, Margherita Maioli

**Affiliations:** 1 Department of Biomedical Sciences, University of Sassari, 07100 Sassari, Italy; scruciani@uniss.it (S.C.); giugarroni21@gmail.com (G.G.); dilettaserra9@gmail.com (D.S.); mmaioli@uniss.it (M.M.); 2 Department of Reparative and Regenerative Medicine, Rinaldi Fontani Institute, 50144 Florence, Italy; vfontani@irf.it (V.F.); ari@irf.it (A.R.); 3 Research Department, Rinaldi Fontani Foundation, 50144 Florence, Italy

**Keywords:** endogenous bioelectrical activity, bioelectrical modulation, REAC technology, dermal fibroblasts, longevity-associated signaling, inflammaging, SIRT1–FOXO–mTOR axis, cytokine signaling

## Abstract

Chronic low-grade inflammation, altered microvascular support, and progressive stress-related cellular dysfunction are major contributors to tissue aging and impaired repair. Dermal fibroblasts are central regulators of these processes because they integrate cytokine-related signaling, redox balance, and extracellular matrix homeostasis. Increasing evidence indicates that endogenous bioelectrical activity may influence these cellular functions by shaping upstream regulatory conditions linked to downstream molecular responses. In the present study, we investigated the molecular effects of the Radio Electric Asymmetric Conveyer Anti-Inflammatory Cellular Treatment delivered under Inside Blue Zone conditions (REAC ACT-IBZ) in human dermal fibroblasts (HFF1). Cells were exposed to nine standardized treatment sessions, and molecular changes were assessed by RT-qPCR, ELISA, and immunofluorescence analysis complemented by supportive semi-quantitative fluorescence intensity assessment. REAC ACT-IBZ exposure was associated with increased SIRT1 and VEGF expression and with transcriptional modulation of selected cytokine-related genes, including IL-1α, IL-1β, IL-2, and IL-8. Immunofluorescence analysis, complemented by supportive semi-quantitative fluorescence intensity assessment, showed a pattern consistent with increased FOXO1 and SIRT1 staining and reduced mTOR staining in treated cells. Overall, these findings identify a molecular profile associated with REAC ACT-IBZ exposure in human dermal fibroblasts, involving stress-response regulators, angiogenesis-related signaling, and selective cytokine-related transcriptional changes. Within the limits of the present in vitro model, the data support the view that endogenous bioelectrical modulation may interact with molecular networks relevant to tissue homeostasis and inflammaging.

## 1. Introduction

Chronic low-grade inflammation represents a major biological driver of tissue degeneration and age-related functional decline [1,2]. Together with impaired angiogenesis and progressive cellular senescence, chronic inflammation contributes to defective tissue repair and microvascular dysfunction across multiple organs [3,4]. These processes are increasingly recognized as interconnected components of inflammaging rather than independent pathological events [1].

Dermal fibroblasts play a central role in orchestrating these age- and inflammation-associated alterations [4,5]. Beyond their structural function in extracellular matrix maintenance, fibroblasts regulate cytokine signaling, redox balance, and microvascular support, thereby shaping tissue homeostasis and tissue remodeling processes [6]. Fibroblast dysfunction has been implicated in multiple pathological contexts including aging-related skin degeneration, chronic inflammatory disorders, fibrosis, and impaired wound healing, highlighting these cells as key regulators of tissue adaptive responses or degeneration [5,7,8,9,10].

While fibroblast behavior has traditionally been interpreted primarily through biochemical signaling pathways, increasing evidence indicates that endogenous bioelectrical activity represents an additional regulatory layer capable of influencing cellular organization and gene expression [11,12]. Bioelectrical signaling can regulate cell proliferation, migration, differentiation, and metabolic responses by coordinating multiple downstream molecular pathways [12,13]. Alterations in these bioelectrical regulatory networks have therefore been proposed as contributors to dysfunctional cellular states associated with chronic inflammation, tissue degeneration, and aging-related processes [11].

In this context, exposure to asymmetrically conveyed radio-electric fields has been reported to interact with endogenous bioelectrical activity and influence cellular behavior in several experimental models [14,15,16,17]. Rather than targeting individual molecular pathways directly, such approaches have been proposed to influence upstream regulatory conditions that coordinate complex cellular responses through bioelectrical signaling mechanisms [18]. However, the molecular configurations associated with this type of bioelectrical modulation in dermal fibroblasts remain incompletely characterized.

Several interconnected molecular hubs integrate stress adaptation, inflammation, and cellular aging in fibroblasts. Sirtuin 1 (SIRT1) plays a key role in regulating redox homeostasis and inflammatory tone through the deacetylation of transcriptional regulators such as FOXO proteins and through interaction with the mammalian target of rapamycin (mTOR) pathway [19,20,21,22]. The SIRT1–FOXO–mTOR signaling axis is widely recognized as a central component of cellular stress adaptation and longevity-associated molecular regulation [23]. In parallel, angiogenesis-related pathways mediated by vascular endothelial growth factor (VEGF) contribute to microvascular integrity and tissue repair processes [24,25], while redox-sensitive enzymes such as NADPH oxidase 4 (NOX4) regulate angiogenic signaling and fibroblast activation during tissue remodeling [26,27].

Inflammatory cytokines further modulate fibroblast-mediated tissue responses [28]. While persistent or uncontrolled cytokine signaling contributes to tissue damage [29], controlled and context-dependent activation of inflammatory mediators plays an essential role in early tissue repair and regeneration [30]. Cytokines such as IL-1 family members, IL-2, and IL-8 participate in immune–stromal communication and extracellular matrix remodeling processes, highlighting the complex role of inflammatory signaling in fibroblast biology [31,32]. At the same time, the biological meaning of cytokine modulation depends on context, timing, and association with broader molecular patterns, rather than on directionality alone.

In the present study, we investigated whether modulation of endogenous bioelectrical activity delivered through the Radio Electric Asymmetric Conveyer Anti-Inflammatory Cellular Treatment protocol under Inside Blue Zone conditions (REAC ACT-IBZ) is associated with coordinated molecular changes in human dermal fibroblasts. By examining the regulation of longevity-associated signaling pathways, angiogenesis-related factors, and cytokine expression, we aimed to characterize the molecular profile associated with bioelectrical modulation in fibroblast cellular systems relevant to tissue homeostasis and inflammaging. More specifically, we sought to determine whether REAC ACT-IBZ exposure was associated with a recognizable molecular pattern involving stress-response regulators, angiogenesis-related signaling, and selective cytokine-related transcriptional changes in an in vitro fibroblast model.

## 2. Materials and Methods

### 2.1. Cell Culturing Conditions

Human foreskin fibroblasts (HFF1, ATCC^®^ SCRC-1041™) [33] were obtained from the American Type Culture Collection (ATCC, Manassas, VA, USA) and cultured in Dulbecco’s Modified Eagle’s Medium (DMEM; Life Technologies, Carlsbad, CA, USA) supplemented with 10% fetal bovine serum (FBS; Life Technologies), 2 mM L-glutamine (Euroclone, Milan, Italy), and 1% penicillin/streptomycin (Euroclone, Milan, Italy).

Cells were used at passages 10 and seeded at a density of approximately 2.6 × 10^4^ cells/cm^2^. All treatments were delivered using a medical device based on Radio Electric Asymmetric Conveyer (REAC) technology (BENE mod. 110, ASMED, Scandicci, Italy). Treatment parameters are pre-set by the device and cannot be modified by the operator, ensuring reproducibility of exposure conditions. Although the carrier frequency of the device can be specified as 2.4 GHz, conventional physical descriptors such as emitted power, field intensity at the source, or nominal field characteristics do not adequately represent the biologically relevant interaction underlying REAC exposure. In this system, the emitted signal is not intended to act as a conventional direct energetic stimulus, but to generate asymmetrically conveyed radio-electric conditions designed to interact with endogenous bioelectrical activity. Therefore, the biological effect cannot be meaningfully inferred from source-level emission parameters alone. For reproducibility, the present study reports the operational exposure conditions under experimental control, namely the treatment protocol, number and duration of sessions, inter-session interval, incubation conditions, and the use of preset, operator-independent device parameters.

REAC ACT-IBZ exposure consisted of nine consecutive standardized 30-min sessions administered under controlled incubation conditions (37 °C, 5% CO_2_) at 13-h intervals.

Control cultures were maintained under identical environmental and handling conditions but without exposure to the asymmetrically conveyed radio-electric field, serving as the baseline for comparison. No dedicated sham-device condition was included in the present experimental design.

Each experiment was performed in at least three independent biological replicates to ensure the robustness and reproducibility of the experimental observations. Where indicated in the figure legends, plotted values derive from pooled analytical observations obtained from independent experiments.

The use of device-pre-set parameters ensured standardized exposure conditions across all experimental replicates.

### 2.2. Gene Expression Analysis

Total RNA was extracted from HFF1 cells using the RNeasy Mini Kit (Qiagen, Hilden, Germany) immediately after the 9th (final) REAC ACT-IBZ session and quantified using a NanoDrop One/OneC Microvolume UV–Vis spectrophotometer (Thermo Fisher Scientific, Grand Island, NY, USA). Real-time quantitative PCR (RT-qPCR) was performed using the Luna^®^ Universal One-Step RT-qPCR Kit (New England Biolabs, Ipswich, MA, USA) in a CFX Thermal Cycler (Bio-Rad, Hercules, CA, USA).

Target Ct values for each sample were normalized to GAPDH, used as the reference gene. Relative gene expression levels were calculated using the 2^−ΔΔCt^ method and expressed as fold change relative to untreated control cells. Primers were obtained from Thermo Fisher Scientific (Grand Island, NY, USA).

All experiments were performed using at least three independent biological replicates (*n* ≥ 3), and each RT-qPCR reaction was conducted in technical triplicates to ensure measurement reliability.

### 2.3. ELISA Assays

HFF1 culture supernatants were collected from untreated control cells and from treated cells immediately after the 9th (final) REAC ACT-IBZ session. The concentrations of IL-6, IL-10, and TNF-α were determined using streptavidin-HRP conjugated ELISA systems: Human IL-6 Mini ABTS ELISA Development Kit (PeproTech EC Ltd., London, UK), IL-10 Mini ABTS ELISA Development Kit (PeproTech EC Ltd., London, UK), and Human TNF-α Mini TMB ELISA Development Kit (PeproTech EC Ltd., London, UK), respectively.

Standard curves were generated according to the manufacturer’s instructions for each cytokine analyzed. All samples were measured in duplicate technical replicates, and cytokine concentrations were expressed as mean ± standard deviation (SD).

Each experiment was conducted using at least three independent biological replicates.

### 2.4. Immunofluorescence Analysis

Immunofluorescence analysis was performed to evaluate the expression of FOXO1, SIRT1, and mTOR proteins. Immediately after the 9th (final) REAC ACT-IBZ session, cells were fixed for 30 min at room temperature with 4% paraformaldehyde (Sigma-Aldrich, Darmstadt, Germany) and permeabilized with 0.1% Triton X-100 in PBS (Thermo Fisher Scientific, Grand Island, NY, USA) for 1 h at room temperature under gentle agitation.

Cells were then incubated in blocking solution consisting of 4% bovine serum albumin (BSA) and 0.1% Triton X-100 in PBS for 1 h at room temperature. Subsequently, cells were incubated overnight at 4 °C with primary antibodies against FOXO1, SIRT1, and mTOR under gentle agitation. The following primary antibodies were used: anti FOXO1 (C29H4) Rabbit Monoclonal Antibody (Cell Signaling, Danvers, MA, USA, #2880, dilution 1:200); anti SIRT1 (1F3) Mouse Monoclonal Antibody (Cell Signaling, Danvers, MA, USA, #8469, dilution 1:100); and anti mTOR antibody (Abcam, Cambridge, UK, #ab2732, dilution 1:100).

After three washes in PBS (5 min each), cells were incubated with fluorescence-conjugated secondary antibodies (Life Technologies, Carlsbad, CA, USA) for 1 h at 37 °C in the dark. Nuclei were counterstained with 1 µg/mL 4′,6-diamidino-2-phenylindole (DAPI) (Thermo Fisher Scientific, Grand Island, NY, USA).

Fluorescence images were acquired using a fluorescence microscope (Thunder Imager DMi8, Leica, Nussloch, Germany).

Each experiment was performed in at least three independent biological replicates. Immunofluorescence imaging was used as a qualitative assessment complemented by supportive semi-quantitative fluorescence intensity analysis of FOXO1, SIRT1, and mTOR expression patterns. This analysis was intended as complementary evidence and not as a stand-alone quantitative protein assay.

### 2.5. Statistical Analysis

Statistical analyses were performed using GraphPad Prism 9.0 (GraphPad Software, San Diego, CA, USA). Comparisons between control and REAC ACT-IBZ-treated HFF1 cells were performed using either unpaired two-tailed *t*-tests or Mann–Whitney U tests, depending on data distribution, which was assessed using the Shapiro–Wilk test. A *p* value < 0.05 was considered statistically significant. Significance thresholds were defined as * *p* < 0.05, ** *p* < 0.01, *** *p* < 0.001, and **** *p* ≤ 0.0001.

Given the pathway-oriented nature of the molecular analysis and the limited number of biological replicates, statistical results were interpreted together with effect directionality and consistency across independent experiments. No formal correction for multiple comparisons was applied.

## 3. Results

To investigate the molecular effects associated with REAC ACT-IBZ treatment in human dermal fibroblasts, gene expression analyses were performed focusing on regulators of longevity-associated signaling, angiogenesis, and inflammatory pathways.

RT-qPCR analysis revealed a significant increase in SIRT1 expression in treated cells compared with untreated controls (*p* < 0.05). A concomitant increase in VEGF expression was also observed, indicating modulation of pathways involved in angiogenesis and cellular adaptive responses.

In addition, the expression of several cytokine-related genes was modulated following REAC ACT-IBZ treatment. Specifically, IL-1α, IL-1β, IL-2, and IL-8 showed increased transcriptional levels compared with control cells, whereas TNF-α expression did not show significant changes.

Immunofluorescence analysis further demonstrated increased protein expression of FOXO1 and SIRT1 together with reduced mTOR levels in treated fibroblasts compared with untreated controls.

Taken together, these results indicate that REAC ACT-IBZ treatment is associated with coordinated modulation of molecular pathways involved in cellular stress-response regulation, angiogenesis-related signaling, and cytokine-mediated cellular communication.

### 3.1. Effect of REAC ACT-IBZ on Selected Molecular Markers in Human Dermal Fibroblasts

**Figure 1:**
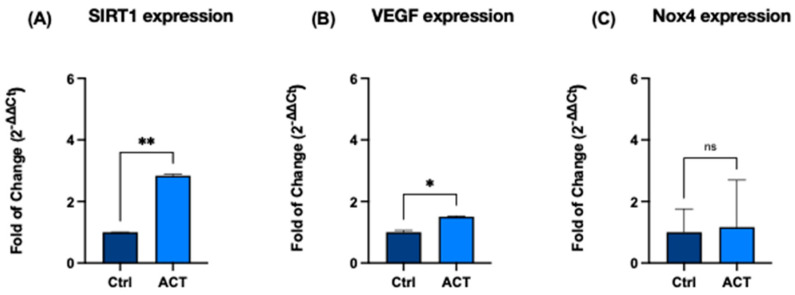
Effects of REAC ACT-IBZ treatment on the expression of selected molecular markers in HFF1 fibroblasts. mRNA expression levels of SIRT1 (**A**), VEGF (**B**), and NOX4 (**C**) were evaluated by RT-qPCR following REAC ACT-IBZ exposure and compared with untreated controls. Gene expression levels were normalized to GAPDH and expressed as fold change using the 2^-ΔΔCt^ method, with control values set to 1. Data are presented as mean ± SD (*n* = 3 analytical values derived from three independent experiments). Statistical significance was determined relative to control samples (* *p* ≤ 0.05; ** *p* ≤ 0.01). ns indicates not significant.

HFF1 fibroblasts were exposed to nine 30-min sessions of REAC ACT-IBZ, and the expression of genes associated with longevity-associated, angiogenic, and redox-related pathways was evaluated by RT-qPCR. A significant upregulation of SIRT1 mRNA expression was observed following REAC ACT-IBZ treatment, with a 2.7-fold increase compared with untreated controls (*p* < 0.05; Figure 1A).

Similarly, VEGF mRNA expression was significantly increased in REAC ACT-IBZ-treated fibroblasts compared with controls (1.5-fold increase, *p* < 0.05; Figure 1B), indicating modulation of pathways involved in angiogenesis-related cellular responses.

Analysis of NOX4 mRNA expression showed a modest increase following REAC ACT-IBZ treatment (control = 1.0; ACT-IBZ = 1.1); however, this difference did not reach statistical significance (*p* = 0.08; Figure 1C).

### 3.2. Effect of REAC ACT-IBZ on Cytokine-Related Gene Expression

To assess the impact of REAC ACT-IBZ treatment on cytokine-related signaling, the expression of IL-1α, IL-1β, IL-2, and IL-8 was evaluated by RT-qPCR.

**Figure 2:**
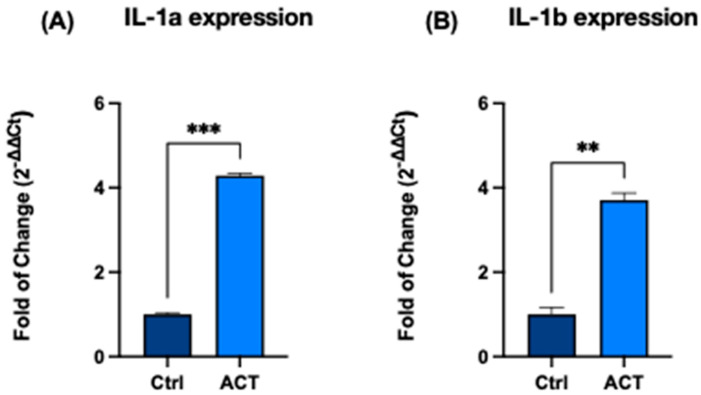

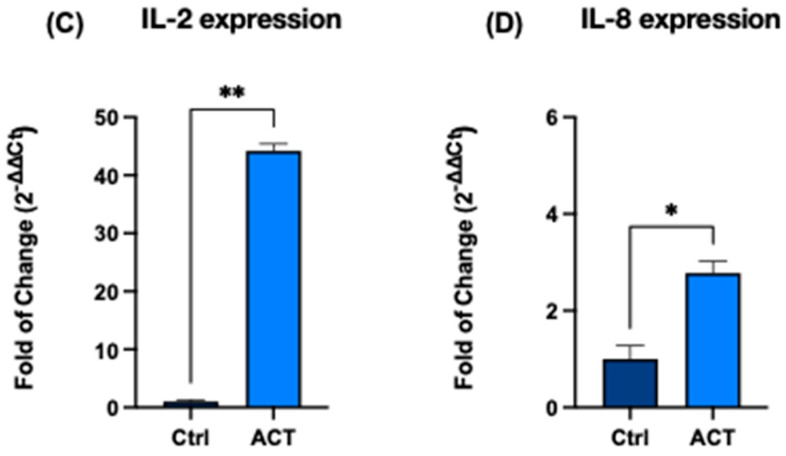
Effects of REAC ACT-IBZ treatment on cytokine-related gene expression in HFF1 fibroblasts. mRNA expression levels of IL-1α (**A**), IL-1β (**B**), IL-2 (**C**), and IL-8 (**D**) were evaluated by RT-qPCR following REAC ACT-IBZ exposure and compared with untreated controls. Gene expression levels were normalized to GAPDH and expressed as fold change using the 2^−ΔΔCt^ method, with control values set to 1. Data are presented as mean ± SD (*n* = 3 analytical values derived from three independent experiments). Statistical significance was determined relative to control samples (* *p* ≤ 0.05; ** *p* ≤ 0.01; *** *p* ≤ 0.001).

Both IL-1α and IL-1β showed significant upregulation following REAC ACT-IBZ exposure, with increases of 4.2-fold and 3.7-fold, respectively, compared with untreated controls (*p* < 0.01; Figure 2A,B).

A marked increase in IL-2 expression was also observed, reaching 42-fold relative to control cells (*p* < 0.001; Figure 2C).

In addition, IL-8 expression was significantly increased (2.6-fold vs. control, *p* < 0.05; Figure 2D).

Overall, REAC ACT-IBZ treatment was associated with a consistent transcriptional modulation of cytokine-related genes in human dermal fibroblasts. Given the absence of dedicated functional assays, these findings should be interpreted as evidence of selective cytokine-related transcriptional modulation rather than as direct evidence of reparative activity.

### 3.3. Effect of REAC ACT-IBZ on Cytokine Release

**Figure 3:**
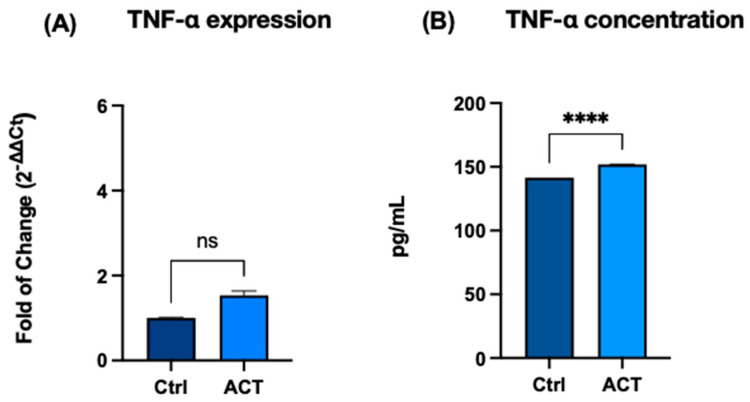
Effects of REAC ACT-IBZ treatment on TNF-α expression in HFF1 fibroblasts. TNF-α mRNA levels (**A**) and protein concentrations (**B**) were evaluated following REAC ACT-IBZ exposure and compared with untreated controls. mRNA levels were normalized to GAPDH and expressed as fold change using the 2^−ΔΔCt^ method, with control values set to 1 (mean ± SD; *n* = 3 analytical values derived from three independent experiments). Protein concentrations were determined by ELISA. TNF-α mRNA expression did not show statistically significant differences between REAC ACT-IBZ-treated and control cells, whereas TNF-α protein concentration was significantly increased in treated cells (**** *p* ≤ 0.0001). ns indicates not significant.

**Figure 4:**
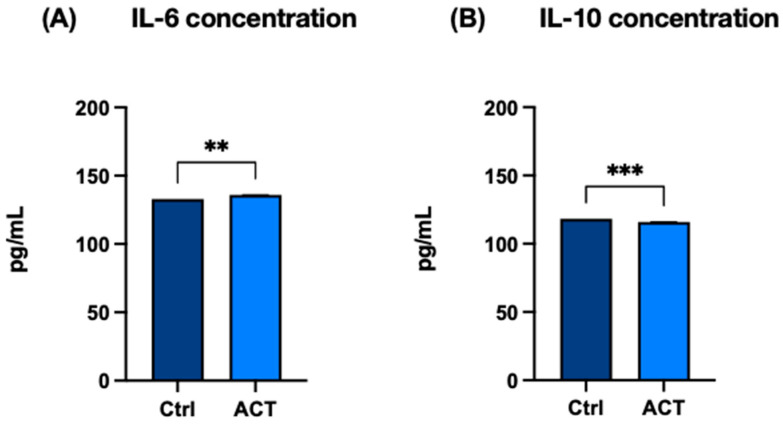
Effects of REAC ACT-IBZ treatment on cytokine secretion in HFF1 fibroblasts. Protein concentrations of IL-6 (**A**) and IL-10 (**B**) were measured by ELISA in culture supernatants collected after REAC ACT-IBZ exposure and compared with untreated controls. Data are presented as mean ± SD (*n* = 3 analytical values derived from three independent experiments). IL-6 protein levels were significantly increased in REAC ACT-IBZ-treated cells compared with controls (** *p* ≤ 0.01), whereas IL-10 protein levels were significantly reduced (*** *p* ≤ 0.001).

To determine whether transcriptional changes were associated with altered cytokine secretion, the protein levels of TNF-α, IL-6, and IL-10 were quantified in culture supernatants by ELISA. TNF-α expression did not show significant changes at the mRNA level following REAC ACT-IBZ treatment (Figure 3A). Conversely, a significant increase in TNF-α protein concentration was observed in culture supernatants (Figure 3B). Similarly, IL-6 secreted levels were significantly increased (Figure 4A), whereas IL-10, an anti-inflammatory cytokine, showed a slight but statistically significant reduction compared with control samples (Figure 4B). Overall, REAC ACT-IBZ treatment was associated with selective changes in cytokine secretion under the experimental conditions analyzed. These findings suggest that REAC ACT-IBZ induces an early and transient pro-inflammatory activation of fibroblasts, potentially promoting a regenerative response.

### 3.4. Integrated Summary of Molecular Changes Associated with REAC ACT-IBZ Treatment

The combined effects of REAC ACT-IBZ treatment on longevity-associated, angiogenic, redox-associated, and cytokine-related genes are summarized in Table 1. Significant modulation was observed for SIRT1, VEGF, IL-1α, IL-1β, IL-2, and IL-8, while NOX4 showed a modest but directionally consistent increase that did not reach statistical significance. TNF-α expression remained unchanged under the experimental conditions analyzed.

These results summarize the molecular expression patterns observed following REAC ACT-IBZ treatment in human dermal fibroblasts.

The consistent directionality of the observed molecular changes across independent experiments further supports the internal coherence of the REAC ACT-IBZ-associated molecular modulation observed in dermal fibroblasts.

### 3.5. Effect of REAC ACT-IBZ Treatment on SIRT1–FOXO–mTOR Signaling

**Figure 5:**
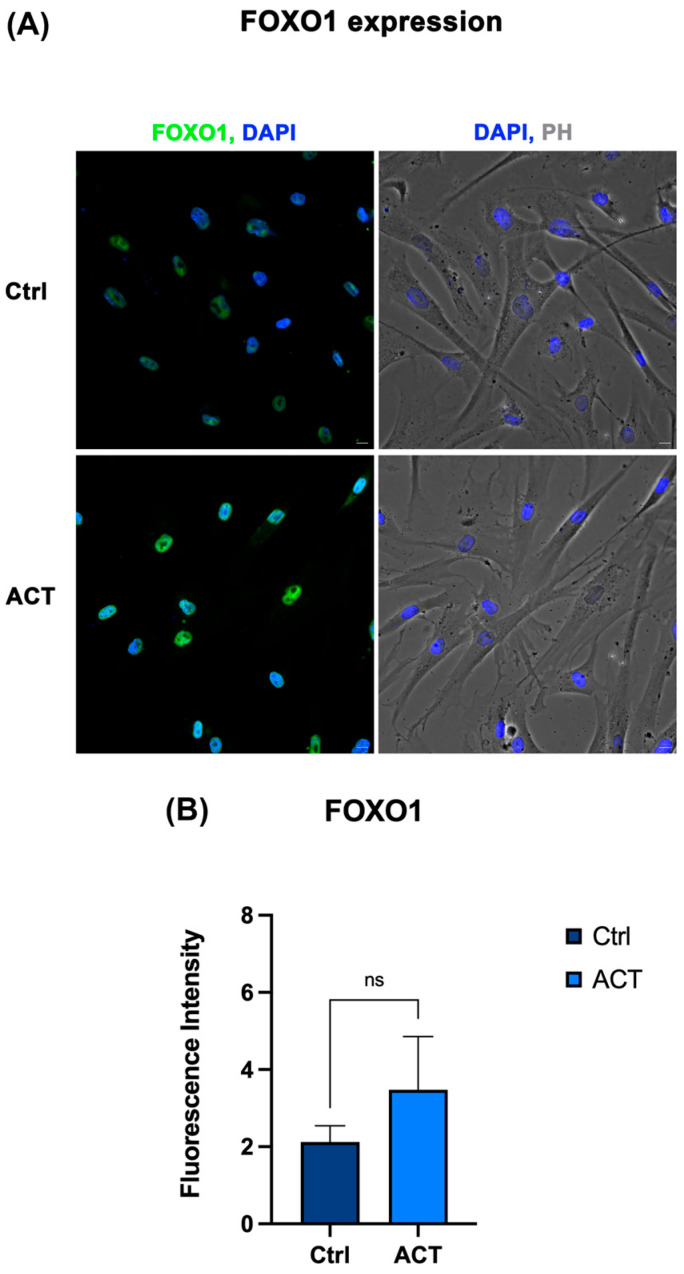
Effects of REAC ACT-IBZ treatment on FOXO1 expression in HFF1 fibroblasts. (**A**) Immunofluorescence analysis of FOXO1 expression in HFF1 fibroblasts following REAC ACT-IBZ treatment compared with untreated controls. Nuclei were counterstained with DAPI (blue). Scale bars: 40 μm. Images are representative of at least three independent experiments. (**B**) Supportive semi-quantitative analysis of FOXO1 fluorescence intensity in control and REAC ACT-IBZ-treated HFF1 fibroblasts. Fluorescence intensity was calculated using ImageJ 1.54s. Data are expressed as mean ± SD. ns indicates not significant.

**Figure 6:**
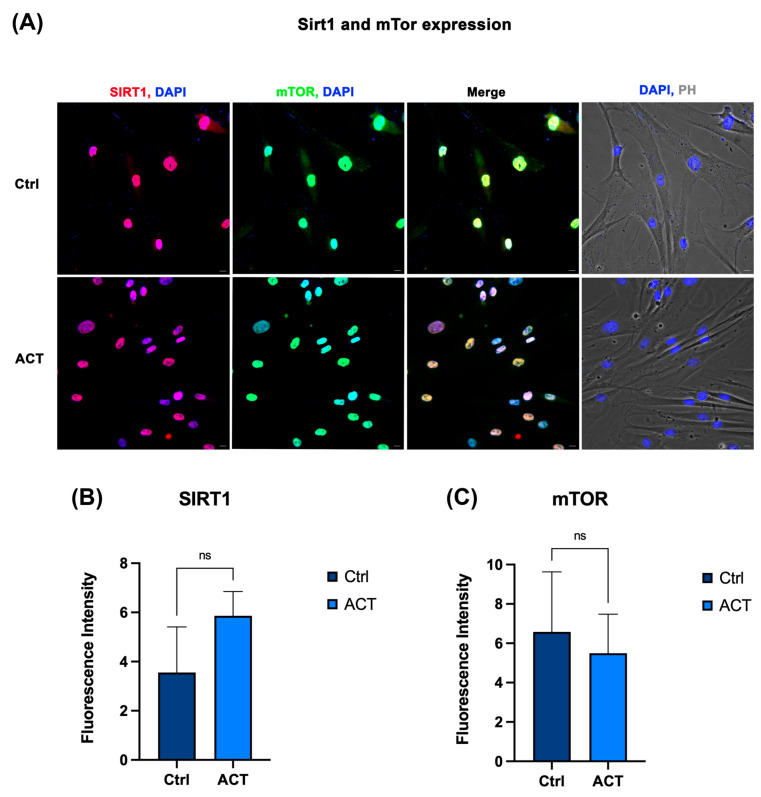
Effects of REAC ACT-IBZ treatment on SIRT1 and mTOR expression in HFF1 fibroblasts. (**A**) Immunofluorescence analysis of SIRT1 and mTOR protein expression in HFF1 fibroblasts following REAC ACT-IBZ treatment compared with untreated controls. Nuclei were counterstained with DAPI (blue). Scale bars: 40 μm. Images are representative of at least three independent experiments. (**B**) Supportive semi-quantitative analysis of SIRT1 fluorescence intensity in control and REAC ACT-IBZ-treated HFF1 fibroblasts. (**C**) Supportive semi-quantitative analysis of mTOR fluorescence intensity in control and REAC ACT-IBZ-treated HFF1 fibroblasts. Fluorescence intensity was calculated using ImageJ 1.54s. Data are expressed as mean ± SD. ns indicates not significant.

Immunofluorescence analysis, complemented by supportive semi-quantitative fluorescence intensity assessment, was performed to evaluate the protein expression of FOXO1, SIRT1, and mTOR in HFF1 fibroblasts following REAC ACT-IBZ treatment. Fluorescence patterns were consistent across independent experiments. FOXO1 expression appeared increased in REAC ACT-IBZ-treated fibroblasts compared with untreated control cells (Figure 5). SIRT1 staining showed a similar increase in treated cells, whereas mTOR signal appeared reduced compared with controls (Figure 5 and Figure 6). Semi-quantitative fluorescence intensity analysis of the acquired images showed a pattern consistent with these qualitative observations, supporting increased FOXO1 and SIRT1 fluorescence signals together with reduced mTOR fluorescence signals in REAC ACT-IBZ-treated cells compared with untreated controls.

These observations are consistent with modulation of molecular regulators associated with cellular stress-response and longevity-related signaling pathways. Because immunofluorescence was used here as a qualitative imaging approach complemented by supportive semi-quantitative fluorescence intensity analysis, these findings should be interpreted as descriptive evidence of a protein expression pattern consistent with the observed molecular profile, rather than as stand-alone quantitative proof of pathway activation.

Overall, the molecular changes observed following REAC ACT-IBZ treatment reveal a coordinated pattern involving regulators of cellular stress adaptation, longevity-associated signaling, and cytokine-mediated communication. The simultaneous upregulation of SIRT1 and increased FOXO1 signal, together with reduced mTOR signal, indicates modulation of a signaling network widely associated with metabolic regulation and cellular resilience. In parallel, the increase in VEGF expression and the selective transcriptional modulation of cytokines suggest engagement of molecular pathways involved in angiogenesis-related responses and immune–stromal communication. These results therefore describe a coherent molecular profile associated with REAC ACT-IBZ treatment in human dermal fibroblasts.

## 4. Discussion

The present study characterizes molecular changes associated with exposure to upstream endogenous bioelectrical modulation in human dermal fibroblasts, revealing coordinated regulation of pathways involved in longevity-associated signaling, angiogenesis, redox balance, and immune–stromal signaling. Rather than reflecting isolated molecular alterations, these findings suggest that REAC ACT-IBZ exposure is associated with a coordinated molecular configuration involving stress-response regulation and selective cytokine-related signaling.

One of the most prominent observations is the significant upregulation of SIRT1, together with increased FOXO1 expression and concomitant downregulation of mTOR. This molecular configuration reflects modulation of the SIRT1–FOXO–mTOR axis, a regulatory hub known to integrate redox homeostasis, metabolic adaptation, and cellular senescence [21,23,34,35]. Regulation of this axis has been widely associated with improved cellular stress adaptation and modulation of senescence-associated signaling in fibroblasts and other cell types [22,36].

In the present study, immunofluorescence, complemented by supportive semi-quantitative fluorescence intensity analysis, provided a protein expression pattern consistent with involvement of this signaling axis, but not a stand-alone quantitative demonstration of pathway activation. The coordinated modulation of these components observed in the present study is therefore consistent with the involvement of upstream regulatory mechanisms rather than isolated single-target effects.

In parallel, REAC ACT-IBZ exposure influenced angiogenesis-related and redox-sensitive pathways. The significant increase in VEGF expression, together with the directionally consistent modulation of NOX4, suggests engagement of molecular networks involved in microvascular support and tissue repair [21,23,34,35]. Although NOX4 changes did not reach conventional statistical significance, previous studies indicate that modest variations in NOX4-dependent redox signaling can influence angiogenic responses and fibroblast activation [26,27]. The combined VEGF–NOX4 pattern observed here is therefore consistent with modulation of pathways involved in vascular support rather than isolated pro-angiogenic activation.

A key aspect of this study is the cytokine transcriptional profile associated with REAC ACT-IBZ exposure. Selective upregulation of IL-1α, IL-1β, IL-2, and IL-8 was observed in the absence of increased TNF-α expression. While these cytokines are often broadly classified as pro-inflammatory mediators, increasing evidence indicates that they also participate in immune–stromal communication, extracellular matrix remodeling, and early reparative processes [31,32]. These findings suggest that REAC ACT-IBZ exposure does not trigger a generalized inflammatory response but may instead be associated with a controlled and context-dependent cytokine signaling profile.

However, in the absence of dedicated functional assays, the present data should be interpreted as evidence of selective transcriptional modulation rather than as direct proof of reparative activity. The magnitude of IL-2 upregulation should nevertheless be interpreted with caution, and future studies using independent validation approaches will be useful to confirm the robustness of this observation.

An additional point requiring consideration is the apparent dissociation between cytokine-related transcriptional changes and the limited differences observed in cytokine secretion by ELISA. This discrepancy may reflect temporal uncoupling between gene transcription and extracellular protein accumulation, post-transcriptional or post-translational regulation, differences in protein stability, or the specific sampling window adopted in the present experimental design. Accordingly, the current results support the presence of selective cytokine-related molecular modulation, but not a generalized secretory inflammatory response under the analyzed conditions.

This observation is particularly relevant given that REAC ACT-IBZ is historically defined as an Anti-Inflammatory Cellular Treatment. The present findings suggest that its biological effects may not involve generalized suppression of cytokine signaling but rather modulation of regulatory conditions that allow selective activation of signaling pathways involved in reparative and adaptive responses while limiting chronic inflammatory activation.

Within the limits of the present in vitro model, this interpretation is more consistent with context-dependent regulatory modulation than with a nonspecific inflammatory activation.

Taken together, these findings indicate that REAC ACT-IBZ exposure is associated with coordinated modulation of interconnected molecular signaling networks in dermal fibroblasts, integrating longevity-associated signaling, redox regulation, and immune–stromal communication.

Rather than acting as a direct downstream anti-inflammatory intervention, this pattern suggests that bioelectrical modulation may influence upstream regulatory conditions capable of affecting multiple cellular signaling networks relevant to tissue homeostasis and inflammaging.

Accordingly, these findings provide molecular insight into how endogenous bioelectrical modulation may interact with cellular signaling involved in stress adaptation and age-related inflammatory regulation.

From a broader perspective, these results complement previous experimental evidence suggesting that modulation of endogenous bioelectrical activity may influence cellular signaling dynamics across multiple biological systems.

The present findings provide a molecular framework that is biologically compatible with previous observations in REAC-based experimental and clinical contexts, although no direct clinical extrapolation can be drawn from the current in vitro model.

Future studies integrating functional assays and in vivo models may help to further elucidate how these coordinated molecular changes translate into tissue-level adaptive responses.

## 5. Conclusions

In conclusion, this study shows that exposure to REAC ACT-IBZ is associated with coordinated modulation of molecular pathways in human dermal fibroblasts. The observed changes involve longevity-associated signaling regulators, angiogenesis-related pathways, and cytokine networks relevant to inflammatory regulation and tissue homeostasis.

Rather than demonstrating a direct therapeutic mechanism, these findings provide molecular insight into how modulation of endogenous bioelectrical activity may interact with cellular signaling systems involved in stress adaptation and inflammaging.

Dermal fibroblasts therefore represent a useful cellular model for investigating the relationship between bioelectrical regulatory mechanisms and coordinated molecular responses. Within the limits of the present in vitro design, the data support the identification of a molecular profile associated with REAC ACT-IBZ exposure, while further studies integrating functional assays, temporal analyses, and in vivo models will be necessary to better define the biological significance of these molecular observations.

## Figures and Tables

**Table 1:** Gene expression values are expressed as fold change relative to untreated control (Ctrl) cells. Statistical significance was assessed using unpaired two-tailed *t*-tests or Mann–Whitney U tests, as appropriate. ns indicates not significant.

Marker	Control (Ctrl)	REAC ACT-IBZ	Fold Change	*p*-Value
SIRT1	1.0	2.7	2.7	<0.05
VEGF	1.0	1.5	1.5	<0.05
NOX4	1.0	1.1	1.1	0.08 (ns)
IL-1α	1.0	4.2	4.2	<0.01
IL-1β	1.0	3.7	3.7	<0.01
IL-2	1.0	42.0	42.0	<0.001
IL-8	1.0	2.6	2.6	<0.05
TNF-α	1.0	1.02	1.02	>0.05 (ns)

## Data Availability

The data supporting the findings of this study are available within the manuscript.
